# The Impact of Peptidoglycan Structure on Immune Sensing

**DOI:** 10.3390/toxins18080354

**Published:** 2026-08-20

**Authors:** Sasha Cardozo, Ciaran Skerry

**Affiliations:** Department of Microbiology and Immunology, University of Maryland School of Medicine, Baltimore, MD 21201, USA

**Keywords:** peptidoglycan, remodeling, toxic fragments, immune-sensing, pattern recognition receptors, signaling, bacterial evasion

## Abstract

Peptidoglycan (PGN) is a mesh like polymer that builds and protects the bacterial cell wall. Across and even within species, PGN varies considerably in structure and composition, and this structural diversity shapes how released fragments are detected by the host. The varied structural features of PGN can also dictate its ability to act as a toxin or danger signal. Traditionally, bacterial cell wall components have been classified as toxic, based on their ability to cause extensive host cell damage, but the specific immune responses triggered by distinct PGN structural motifs when they are released into the extracellular space, and their potential direct role as toxins are not yet fully understood. Emerging evidence suggests that PGN is recognized by the host not only through its canonical di-/tripeptides, but also through novel structural motifs that are sensed by host pattern recognition receptors (PRRs). However, the mechanisms underlying the recognition of these structurally diverse fragments and their role in promoting immune activation, bacterial pathogenesis, and immune evasion remain poorly understood. In this review, we describe how structurally distinct muropeptides are synthesized, processed, and selectively sensed by host PRRs, and how differential immune recognition shapes PRR activation, thereby influencing host–pathogen interactions and infection outcomes.

## 1. Introduction

PGN is an essential structural component of the bacterial cell wall, acting as a scaffold for strength, resistance, and survival. Among the many facets of PGN biology, its sensing and recognition by the host immune system continue to be areas of significant interest and ongoing discovery. Structurally, PGN is a polymer consisting of long linear glycan chains crosslinked by peptide subunits, that serves as a key functional and immune-defining feature of both gram-positive and gram-negative bacterial cell walls [1,2]. The emerging literature suggests that PGN is a dynamic molecule that undergoes continuous remodeling during growth, division, and environmental stress [3,4,5].

The biosynthesis of PGN occurs in three main stages, starting with precursor synthesis and peptide addition in the cytoplasm, followed by lipid-linked transport across the inner membrane, sequential assembly into Lipid I and Lipid II, and finally cross-linking reactions in the periplasm [6]. These processing stages are regulated by bacterial enzymes that modify the sugar backbone, peptide stem, and cross-linking bridges, generating structurally diverse fragments [7]. Structural variants of PGN also serve as strategies to evade host lysozymes, exert toxic effects, enhance immunopathology, and promote resistance to antibiotics [8]. While the enzymatic pathways for peptidoglycan remodeling, such as N-deacetylation, O-acetylation, incorporation of non-canonical D-amino acids, and formation of atypical L,D-crosslinks are well characterized in the literature [9,10], the mechanisms by which pathogens harness these structural modifications to blunt host innate immune signaling are still being explored.

PGN is a microbe-associated molecular pattern (MAMP) and can act synergistically with lipoteichoic acid (LTA), a surface-associated glycolipid in gram-positive bacteria eliciting toxic effects on host tissues. This toxicity occurs through PRR-mediated sensing of PGN, activating MAPK signaling and driving iNOS and nitric oxide (NO) production, which in turn upregulates pro-inflammatory cytokines (IL-6, TNF-α, IL-1β), ultimately damaging host cells [11,12,13]. In macrophages, PGN alone stimulates iNOS production and nitric oxide (NO) release through the activation of MAPK pathways [13]. Further, muramyl dipeptide (MDP), the minimal PGN motif, potentiates NO synthesis via NOD2 signaling [14].

Advances in our understanding of the immunostimulatory properties of diverse PGN fragments could inform two potential therapeutic strategies: (1) selectively modulating inflammatory signaling by using specific PGN fragments to limit excessive PRR activation, and (2) incorporating defined PGN motifs into vaccine adjuvants to boost protective immunity. Emerging studies in the field of innate immunity parallel these ideas using LPS pre-stimulation to reprogram innate immunity against lethal systemic inflammation following bacterial infection [15]. Here, we instead examine PGN structure itself as a modulator of PRR signaling, with the overall goal of defining how distinct PGN fragments shape diverse host responses. The information synthesized here serves as a rationale for the use of designer PGN fragments in the development of next-generation host- and pathogen-directed therapeutics.

## 2. Structural Diversity of Peptidoglycan

### 2.1. Core Structural Features of PGN

The PGN backbone comprises alternating MurNAc and GlcNAc carbohydrates anchored to peptide stems by amide bonds formed at the MurNAc d-lactyl group [16,17]. With the exception of certain Mycoplasma species and cell-wall deficient L-form bacteria, virtually all bacterial species characterized to date have a functional PGN layer [18], including members of the PVC superphylum that lack canonical PGN biosynthesis machinery, but retain a physical PGN sacculus [19]. In Gram-positive bacteria, these strands form a thick highly ordered lattice, with NMR studies revealing a densely packed parallel orientation of PGN chains [20].

The PGN peptide stem is typically synthesized as a pentapeptide containing both L- and D- amino acids, most commonly L-Ala-D-Glu-meso-DAP-D-Ala-D-Ala and L-Ala-D-Glu-L-Lys-D-Ala-D-Ala in gram-negative and gram-positive bacteria respectively [21]. The terminal D-Ala is cleaved during transpeptidation, the final enzymatic step in PGN synthesis, in which neighboring peptide stems are covalently linked by DD-transpeptidases and penicillin-binding proteins (PBPs) [22,23].

The proportion of peptide stems that are cross-linked is a major determinant of cell wall rigidity and architecture. Higher cross-link density typically increases wall-stiffness and reduces pore size, whereas lower cross-linking can generate a more permissive network facilitating insertion of newly synthesized components during cell wall growth and remodeling [24]. For example, in *S. aureus* PGN, high inter-peptide cross-link density correlates with increased resistance to mechanical lysis, beta-lactams, host defensins and antimicrobial peptides. Another example is the covalent addition of mycolic acids and arabinogalactan to the PGN in *M. tuberculosis*, which helps increase cell wall density and hydrophobicity. These cell wall adaptations act as a primary barrier to antibiotic penetration, directly contributing to enhanced antibiotic resistance in MRSA and TB infections [25,26,27].

**Figure 1:**
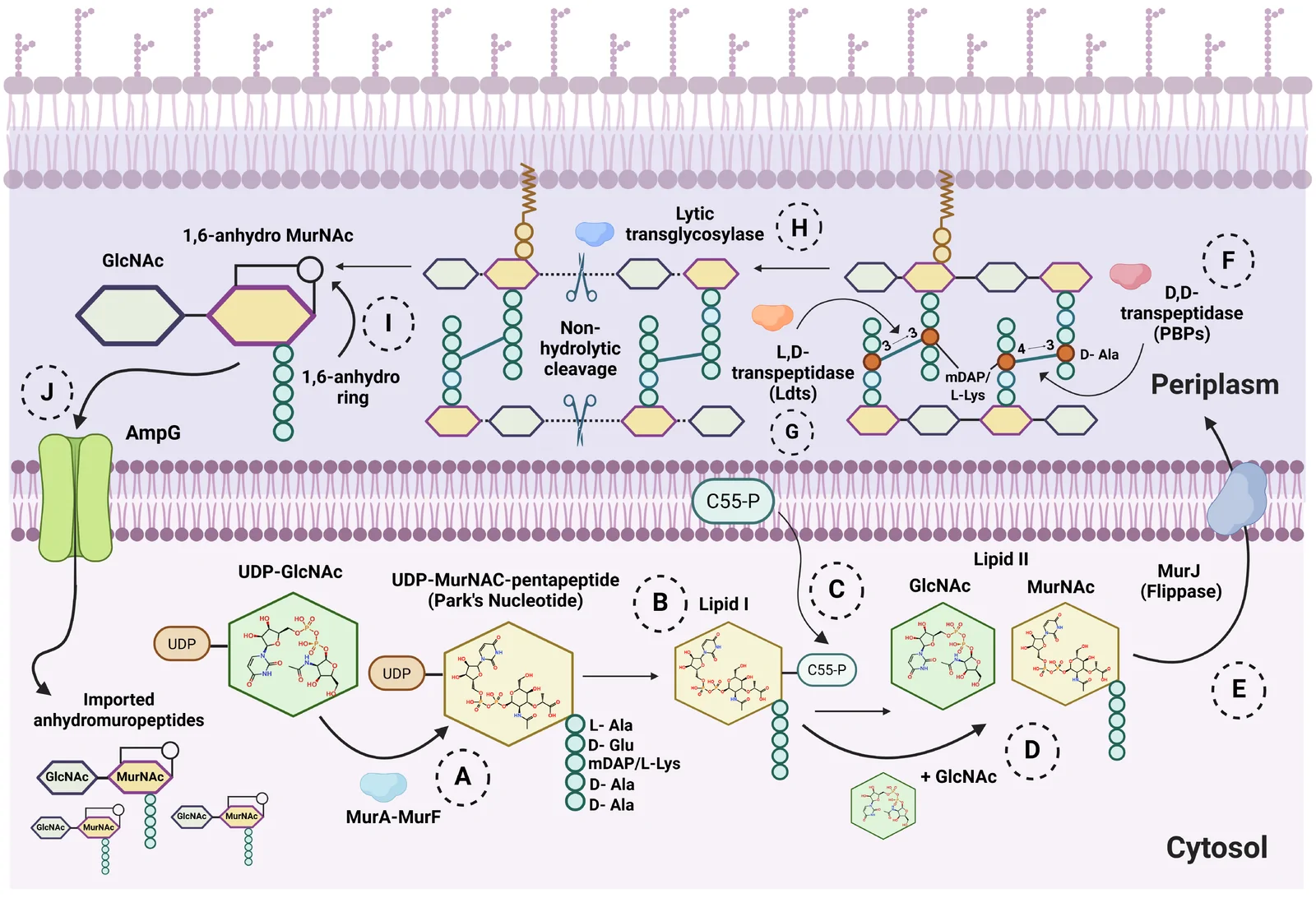
PGN synthesis and turnover. UDP-MurNAc-pentapeptide (Park’s nucleotide) is generated in the cytosol by MurA-MurF enzymes (A) from UDP-GlcNAc. Lipid I (B) is then formed by the coupling of C55-P (C). Addition of GlcNAc (D) generates Lipid II, which is flipped across the inner membrane by MurJ (E). In the periplasm, the GlcNAc-MurNAc-pentapeptide is polymerized into the glycan backbone and cross-linked by D,D-transpeptidases (PBPs) (F) and L,D-transpeptidases (Ldts) (G), forming 4→3 and 3→3 peptide cross-links, respectively. Lytic transglycosylases act on the mature PGN via non-hydrolytic cleavage (H), releasing 1,6-anhydro-MurNAc-containing muropeptides (I), which are imported into the cytosol through the AmpG permease (J) for subsequent processing. [Created in BioRender. Cardozo, S. (2026) https://BioRender.com/4wqhkc1].

In addition to the canonical 4→3 cross-links that join Lys- or DAP-containing stems at position 3 to D-Ala at position 4 in most gram-positive and gram-negative PGN (Figure 1), respectively, *Vibrio cholerae* commonly harbors 3→3 cross-links formed between meso-DAP residues on adjacent peptide stems. These 3→3 cross-links, as shown in Figure 1 are formed by L,D-transpeptidases (Ldts), a class of PGN cross-linking enzymes rather than the canonical PBPs, which exclusively catalyze 4→3 linkages, serving to regulate lytic transglycosylase activity, mediate antibiotic resistance, strengthen the cell wall and adapt to cell envelope stress [28]. In addition to catalyzing atypical 3→3 cross-links, Ldts have also been reported to form 1→3 cross-links in certain species, incorporate non-canonical D-amino acids into the stem peptide, and mediate covalent attachment of outer membrane lipoproteins to PGN [29].

More recently it has been shown that bacteria actively release D-amino acids into the environment during their growth and that these can be incorporated into the PGN of neighboring bacteria and even act as signaling molecules that regulate biofilm formation and sporulation [30]. Together these structural parameters—glycan strand length, crosslink type and density, and stem peptide composition—dictate bacterial growth, survival, and pathogenesis.

### 2.2. PGN Synthesis and Enzymatic Processing

PGN is continually synthesized, remodeled, and degraded as part of normal bacterial physiology. The fragments generated during this turnover reflect a tightly regulated system that accommodates bacterial growth and division and environmental shifts. Rod shaped bacteria insert new glycan strands into their cell walls by first cleaving existing crosslinks and glycan strands, a process that liberates soluble fragments into the periplasm and surrounding environment. This cleavage is carried out by autolysins that generate insertion sites and is spatially constrained by the actin homolog MreB, which organizes PGN synthesis and ensures that new strand generation and cleavage occur synchronously [31,32]. In *E. coli* approximately 40–50% of PGN mass is turned over every generation, making it one of the most metabolically active components of the cell [33].

The stepwise assembly of PGN starts in the cytoplasm with UDP-MurNAc-pentapeptide, a soluble precursor sometimes called Park’s nucleotide after its discoverer James Park [34]. Mur ligases (MurA–MurF) play a critical role in building this precursor from scratch, following which it is linked to the membrane carrier undecaprenyl phosphate (C55-P) to form Lipid I. The subsequent addition of GlcNAc produces Lipid II, a membrane-anchored monomer that is translocated across the inner membrane by flippases, most prominently MurJ in *E. coli* [35]. Once in the periplasmic compartment, PBPs polymerize Lipid II into glycan strands via transglycosylation and crosslink adjacent strands via transpeptidation (Figure 1).

This crosslinked PGN undergoes continuous breakdown to ensure efficient turnover for recycling. PGN turnover is driven by dedicated enzymatic machinery that cleaves the cell wall at specific sites, producing structurally defined fragments. In *E. coli*, and other gram-negative bacteria lytic transglycosylases (LTs) cleave the β-1,4-glycosidic bond between GlcNAc and MurNAc in the periplasm, producing 1,6-anhydromuropeptide monomers characterized by a 1,6 anhydro ring at the reducing end of the MurNAc residue [36] (Figure 1). In contrast to the periplasmic recycling of PGN by LTs observed in most gram-negatives, external amidases contribute to fragment generation in many gram-positive bacteria by hydrolyzing peptide crosslinks at the outer cell wall surface. In *S. aureus* the major autolysin, Atl, comprises an N-acetylmuramyl-L-alanine amidase (AmiA) that removes the stem peptide from MurNAc, creating non-1,6-anhydromuropeptides for downstream glucosaminidase activity [37].

**Figure 2:**
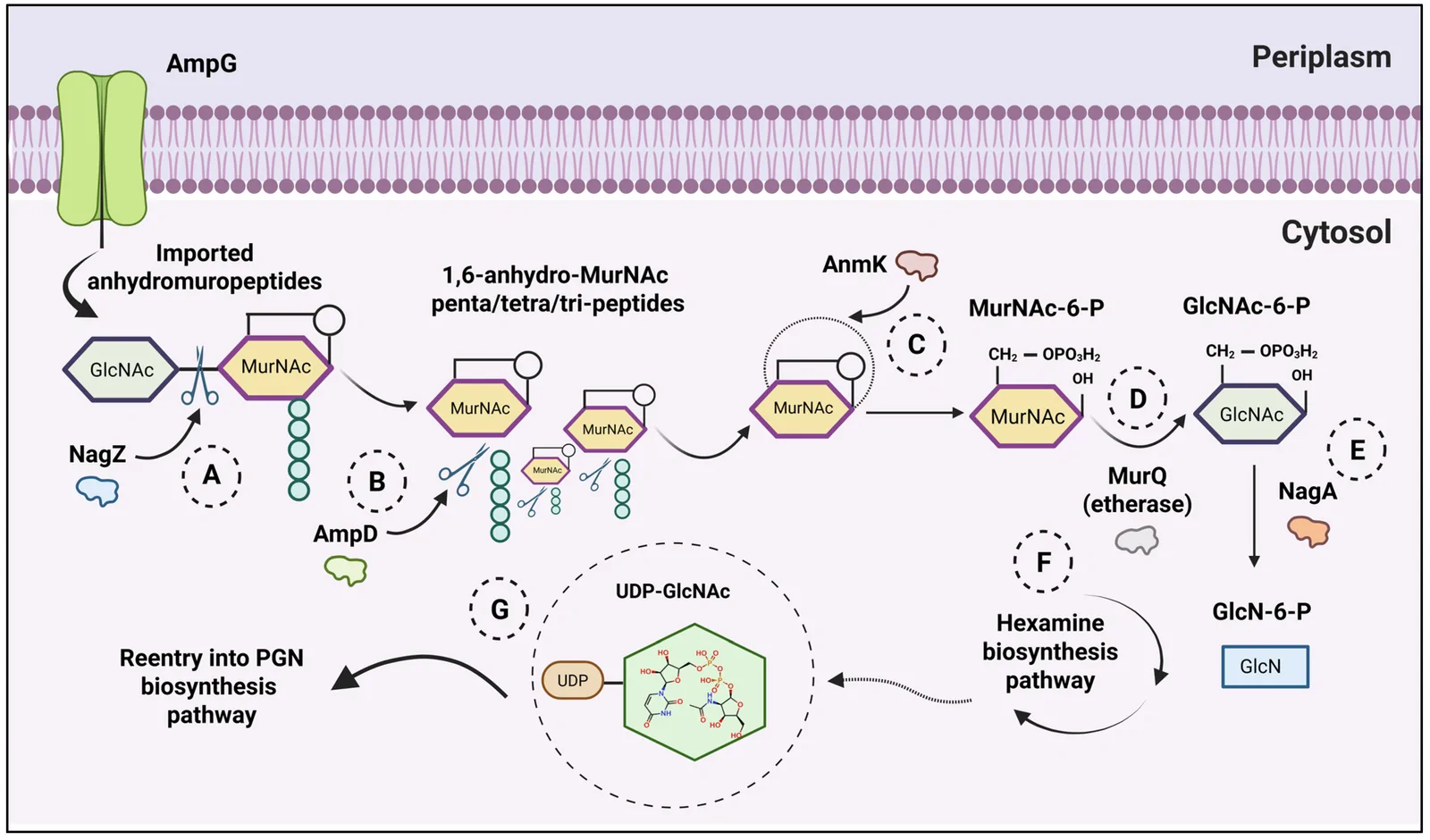
Cytosolic recycling of anhydromuropeptides via the AmpG pathway. 1,6-anhydro-muropeptides are imported through the periplasmic transporter AmpG and sequentially processed in the cytosol: NagZ cleaves the bond between GlcNAc and MurNAc (A) and AmpD removes the stem peptide, resulting in 1,6-anhydro-MurNAc penta/tetra/tripeptides (B). AnmK opens the 1,6-anhydro ring to generate MurNAc-6-phosphate (C), which is converted to GlcNAc-6-phosphate by the etherase MurQ (D). GlcNAc-6-P is then either deacetylated by NagA (E) to GlcN-6-P to be used in the hexosamine biosynthesis pathway (F) or channeled back to UDP-GlcNAc (G) for reentry into the PGN biosynthesis pathway. [Created in BioRender. Cardozo, S. (2026) https://BioRender.com/2nax6u4].

1,6 anhydro PGN monomers are transported into the cytoplasm via the inner membrane permease AmpG, for cytosolic processing (Figure 2). Once inside the cytosol, NagZ, a β-N-acetylglucosaminidase, cleaves the GlcNAc-1,6-anhydroMurNAc disaccharide, generating free GlcNAc and 1,6-anhydroMurNAc residues. AmpD, a cytoplasmic N-acetylmuramyl-L-alanine amidase, cleaves the stem peptide from the glycan, resulting in the release of the intact stem peptide and 1,6-anhydro muropeptides. The liberated stem peptide acts as a source of recyclable D-amino acids, such as mDAP in gram-negatives, which is separately shuttled back into the cytoplasm by the protein MppA together with the transporter OppBCDF, where it is trimmed and reattached to UDP-MurNAc for reuse [38]. As depicted in Figure 2, the stem-free 1,6 anhydroMurNAc is then phosphorylated into MurNAc-6-phosphate by AnmK, an anhydro-N-acetylmuramic acid kinase. Subsequent conversion to GlcNAc-6-phosphate is carried out by MurQ, a hydrolase. NagA, an N-acetylglucosamine-6-phosphate deacetylase then deacetylates the intermediate into GlcN-6-P, which reenters the *de novo* pathway for UDP-GlcNAc and UDP-MurNAc synthesis to ultimately form Lipid I and Lipid II for cell wall assembly [38,39].

### 2.3. Release of Structurally Diverse PGN Fragments

**Figure 3:**
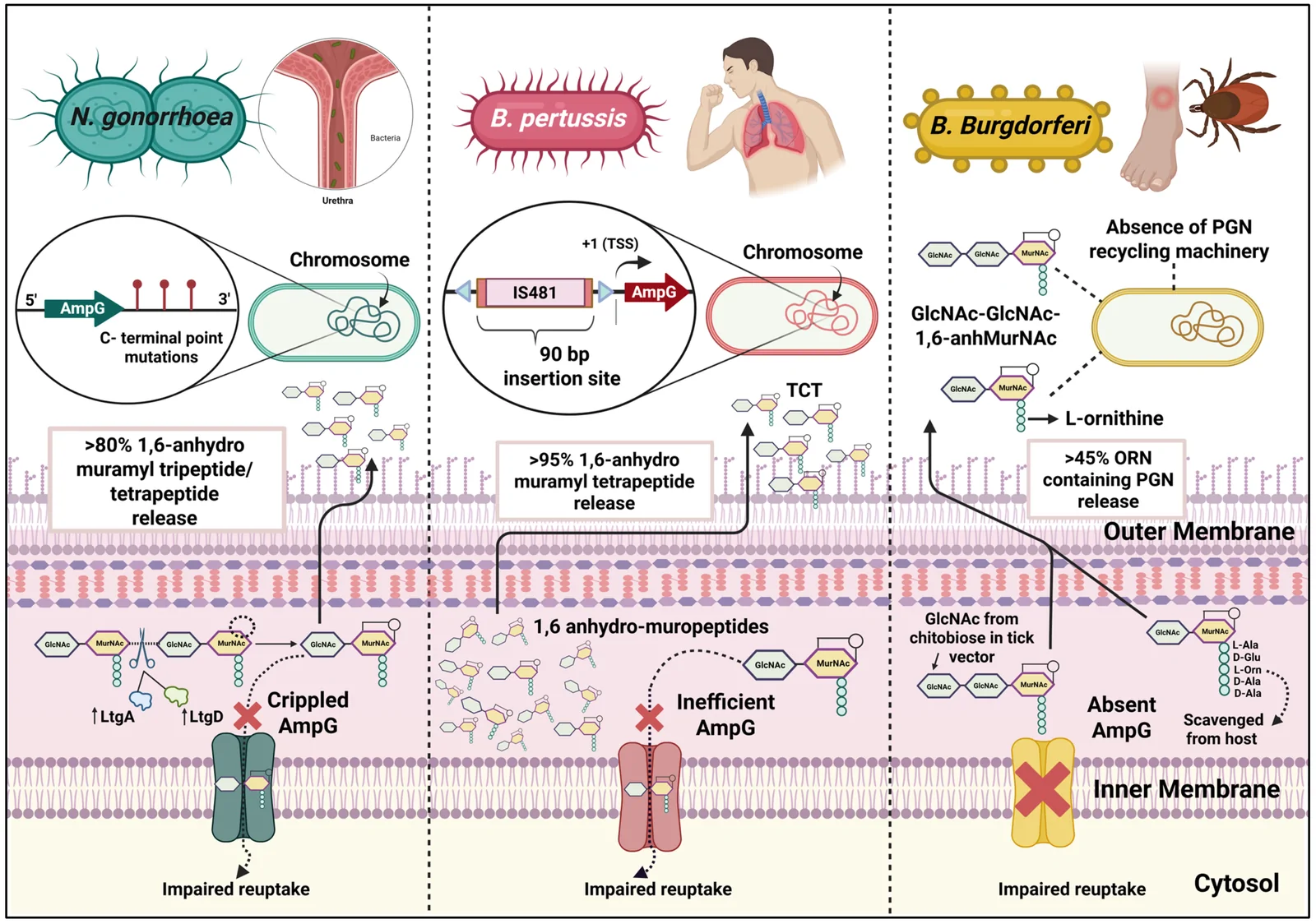
Defects in PGN recycling drive the release of structurally diverse fragments. *N. gonorrhoeae* carries C-terminal point mutations in AmpG that impair its transport function (**left**), and *B. pertussis* harbors an IS481 insertion that reduces AmpG expression (**center**); these defects impair muropeptide reuptake, driving the constitutive release of >80% and >95% 1,6-anhydro muramyl tri-/tetrapeptide (TCT), respectively. *B. burgdorferi* lacks AmpG and the recycling machinery entirely and incorporates host-derived L-ornithine and tick-derived GlcNAc into its PGN, releasing >45% ornithine-containing muropeptides (**right**). Across all three pathogens, defective recycling drives extracellular accumulation of immunostimulatory PGN fragments that have unique downstream signaling implications. [Created in BioRender. Cardozo, S. (2026) https://BioRender.com/d8iu2mw].

Most gram-negative bacteria that actively recycle PGN can reimport the 1,6 anhydro MurNAc fragments generated by LTs through inner membrane permeases as discussed earlier in Section 2.2. However, not all PGN fragments generated during remodeling are retained inside the cell. Some species like *N. gonorrhoeae* and *B. pertussis* have reduced AmpG efficiency, which results in the constitutive release of 1,6 anhydro MurNAc fragments into the extracellular space [40,41] (Figure 3).

Specifically, *N gonorrhoeae* harbors three C-terminal point mutations that cripple its AmpG permease, while *B. pertussis* carries an upstream insertion sequence (IS481) insertion that suppresses AmpG transcription contributing to inefficient PGN recycling [42,43]. The leakage of these 1,6 anhydro PGNs is considered non-canonical, as these fragments are generally intracellular recycling intermediates in other bacteria. Contrastingly, in *P. aeruginosa* AmpG acts both as a stress sensor as well as a transporter, with much broader substrate specificity compared to *E. coli*, importing both anhydromuropeptides and larger fragments that other bacterial permeases cannot process [44,45].

Another non-canonical PGN structure is released by *B. burgdorferi*, characterized by L-ornithine at the third position of its stem peptide (Figure 3), which is the position typically occupied by mDAP or L-lysine in most other bacteria. While L-ornithine as a crosslinking diamino acid has been reported across several bacterial groups including spirochetes, actinomycetes, thermophiles, gliding bacteria, and certain firmicutes, the active extracellular shedding of these L-ornithine-containing PGN fragments is unique to *B. burgdorferi* [46,47,48]. These atypical PGN compositions and structures have important implications for how PRRs sense them to activate downstream signaling responses as will be discussed for some of these non-canonical PGN structures later in this review.

## 3. Immune Sensing of PGN Fragments

The structural features of PGN fragments determine a plethora of physiological processes in the host ranging from immune activation, metabolic programming, autophagy and apoptosis. Findings from seminal studies shed light on the potential use of PGN fragments as targeted adjuvants for vaccine therapeutics. In 1937, Jules Freund and colleagues found that heat-killed mycobacteria suspended in a mineral oil emulsion, now known as Freund’s adjuvant, robustly amplified antibody responses in animals [49]. The immunostimulatory characteristics of this adjuvant were later traced to PGN in 1974, where Ellouz and group demonstrated that the synthetic dipeptide MDP was sufficient to replace killed mycobacteria completely in Freund’s adjuvant formulations [50]. Unlike gut-derived PGN that circulate systemically below the threshold of overt inflammation, pathogen derived PGN is usually presented more locally along with other PAMPs during acute infections driving NF-κB mediated inflammatory activation [51]. These immune responses are orchestrated by a functionally diverse set of PRRs that operate in distinct cellular compartments.

### 3.1. Extracellular PGN Sensing

At the cell surface and in the extracellular space, peptidoglycan recognition proteins (PGRPs) constitute a conserved family of PGN-binding innate immune receptors present in insects and mammals [52]. PGRPs were first identified in silkworm hemolymph as a protein that bound PGN and activated the prophenoloxidase cascade [53]. Mammalian orthologs (PGLYRP 1-4) were subsequently identified in mammals by sequence homology and shown to maintain PGN binding by a conserved muramyl-binding group, despite varied effector functions [52,54]. All four family members have a conserved C-terminal PGN binding domain that is structurally homologous to the bacteriophage T7 lysozyme [55].

**Figure 4:**
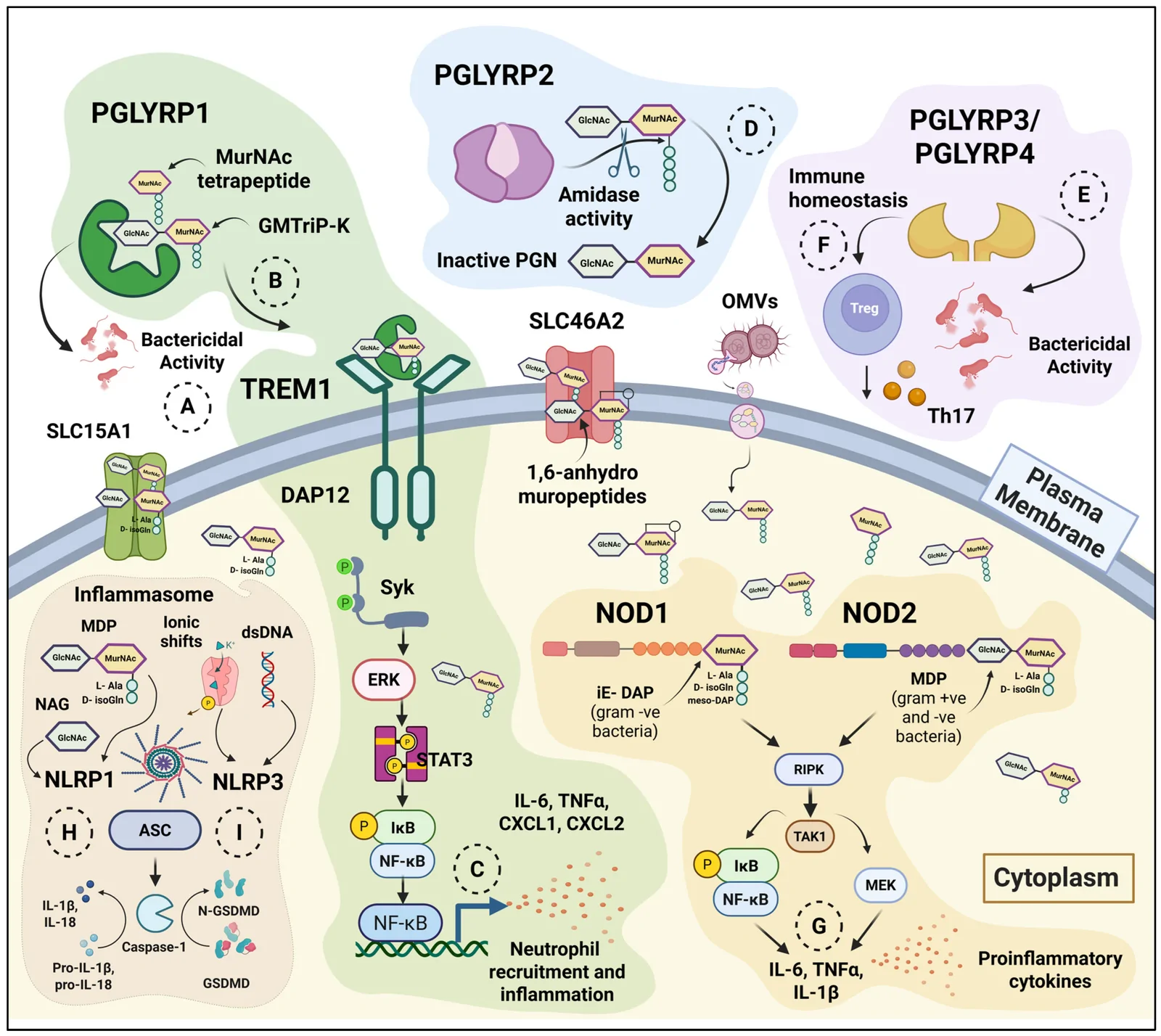
Immune sensing of PGN by intracellular and extracellular PRRs. Muropeptides released from bacterial PGN are recognized by extracellular receptors (PGLYRP1-4), the membrane receptor TREM1/DAP12, and the transporters SLC15A1 and SLC46A2, which deliver fragments into the cytoplasm. PGLYRP1 is both directly bactericidal (A) and a PRR for PGN that is subsequently presented as a PGLYRP1–PGN complex to TREM-1 (B), resulting in TREM-1 activation through Syk-ERK-STAT3 and NF-κB-mediated pro-inflammatory signaling (C). PGLYRP2 is the solo amidase that degrades PGN fragments making them less immunostimulatory (D). PGLYRP3 and PGLYRP4 display bactericidal activity (E) and regulate Treg/Th17 responses contributing to tissue homeostasis (F). OMVs facilitate additional cytosolic transport of PGN fragments making them accessible to intracellular receptors. Cytosolic sensing by NOD1 and NOD2 activates RIPK2-TAK1-NF-κB signaling (G), while MDP and NAG also engage the NLRP1 inflammasome (H). NLRP3 responds to ionic shifts and dsDNA release (I), and both inflammasome proteins activate Caspase-1-mediated IL-1β, IL-18, and Gasdermin-D (GSDMD) release. These host PGN sensing pathways converge on a range of immune responses that drive proinflammatory cytokine release, pyroptosis, and neutrophil recruitment. [Created in BioRender. Cardozo, S. (2026) https://BioRender.com/rfaifwo].

PGLYRP1 is concentrated in neutrophilic granules and is released upon degranulation at infection sites, where it binds PGN to trigger bactericidal activity and downstream immune signaling upon infection [56,57,58]. PGLYRP1-mediated immune activation triggers pro-inflammatory effects in conditions like asthma, dermatitis, psoriasis, and rheumatoid arthritis [59,60,61]. PGLYRP2 is secreted primarily by the liver in humans and is the only mammalian PGLYRP to carry the N-acetylmuramoyl-L-alanine amidase activity that cleaves the lactyl-amide bond between MurNAc and the first stem-peptide residue (L-Ala), uncoupling the PGN backbone (GlcNAc-MurNAc) from the peptide stem [62]. The resulting fragments are comparatively weaker NOD agonists (Figure 4), because they fail to represent the minimal structural motifs required for NOD1 (GlcNAc-MurNAc-tripeptide; GM-triDAP) and NOD2 (MurNAc-L-Ala-D-isoGln; MDP) recognition. While NOD1 is able to sense the free stem peptide (γ-D-Glu-meso-DAP; iE-DAP), it is a weaker agonist compared to the intact GM-triDAP [63].

Extracellularly, PGLYRP1-PGN complexes act as ligands for triggering receptor expressed on myeloid cells (TREM-1), an innate immune-activating receptor that drives downstream pro-inflammatory gene transcription (Figure 4). In this interaction, PGN acts as a scaffold for PGLYRP1 multimerization, enhancing PGLYRP1-mediated TREM-1 activation and inflammatory signaling [64]. PGLYRP1 has also been shown to stimulate microglia in a PGN-independent manner by binding TREM-1 and activating the Syk-Erk1/2-Stat3 signaling pathway to trigger neuroinflammation; however, mutanolysin-treated PGN significantly diminished this effect, highlighting the distinct contribution of PGN in facilitating PGLYRP1-driven immune responses [65]. In contrast PGLYRP2 acts as a negative immune regulator by reducing production of local pro-inflammatory cytokines and PMN infiltration to the hind limbs of MDP-induced arthritic mice [66]. This was also reproduced in a *S. pneumoniae* infection in which PGLYRP2-deficient mice had lower levels of lung pro-inflammatory cytokines [67].

PGLYRP3 and PGLYRP4 are bactericidal proteins (Figure 4) typically secreted as disulphide-linked homo and hetero dimers at epithelial surfaces in the skin and mucosa. They interact with bacterial cell wall peptidoglycan to exert their antibacterial activity [68], and have been previously shown to counteract excessive Th17 production by driving aggregation of Treg cells at sites of chronic inflammation in a contact-dermatitis mouse infection model [60]. Although the latter study (reference [60]) does not directly test whether bacterial PGN itself drives this Treg response, PGLYRP3 and PGLYRP4 promote a less-inflammatory immune state in contrast to the observed pro-inflammatory role of PGLYRP1.

### 3.2. Intracellular PGN Sensing

#### 3.2.1. Nucleotide-Binding Oligomerization Domain Receptors (NODs)

Intracellular PGN sensing is mediated principally by NOD-like receptors (NLRs), of which NOD1 and NOD2 are the best characterized. While NOD1 is more ubiquitously expressed on all cell types, the expression of NOD2 is mostly limited to immune and epithelial cells. Both NOD1 and NOD2 comprise of an N terminal effector binding domain, a central NOD/NACHT domain responsible for nucleotide-dependent oligomerization, and a C-terminal leucine-rich repeat (LRR) domain that mediates ligand sensing [69]. Structurally they differ in their number of caspase activation and recruitment domains (CARDs), with NOD1 containing one and NOD2 containing two CARDs. NOD1 detects γ-D-glutamyl-meso-diaminopimelic acid (iE-DAP), a muropeptide that is most commonly found in gram-negative bacteria, whereas NOD2 binds to MDP (Figure 4), which is common to both gram-positive and gram-negative bacteria [70,71].

Upon PGN binding in the cytosol, the NACHT domain undergoes ATP-dependent conformational changes that expose their CARD domains for downstream signaling [72]. As represented in Figure 4, NOD receptors recruit RIP2, which functions to scaffold downstream signaling mediators to assemble a nodosome through CARD–CARD interactions [73]. NF-κB and MAPK signaling pathways are then initiated downstream of RIPK2 and TAK1- Ser-Thr kinases, inducing inflammation [74]. In addition to muropeptide shed during PGN turnover, PGN precursors generated by bacterial lysis such as UDP-MurNAc-peptides can also be sensed by NOD1 and NOD2, as previously observed upon *C. trachomatis* infection, pointing to distinct NOD receptor signaling profiles depending on whether PGN is derived from pathogen death or normal cell wall turnover [75].

NOD1 has been shown to contribute to immune activation in epithelial cells upon exposure to several gram-negative bacteria including *S. flexneri* and *H. pylori*. Interestingly, NOD1-deficient mice were more susceptible to *H. pylori* and *C. difficile* infections highlighting its role in pathogen recognition and immune defense in vivo [76,77]. NOD2 also activates pro-inflammatory immune responses driving immune defense following infection with *S. pneumoniae*, *L. monocytogenes* and *C. trachomatis* [75,78,79]. Specifically, infection with *S. pneumoniae*, was shown to induce NOD2-dependent CCL2 expression and clearance of bacteria from the lungs 78]. Whereas in the case of *C. trachomatis*, NOD2-dependent PGN recognition was delayed due to the pathogen preferentially accumulating these fragments only in later life stages [75], demonstrating a novel immune evasion strategy and the potential of bacterial biasing of host NOD responses in pathogenesis and disease.

NOD1 and NOD2 also orchestrate different infection-dependent immune outcomes. In a *Streptococcus pyogenes*-induced arthritis infection model, NOD2 deficient mice exhibited reduced joint inflammation and protection against early cartilage damage. This was also replicated in PBMCs treated with bacterial cell wall fragments, wherein cells carrying a NOD2 mutation were impaired in IL-1β and TNFa production. In contrast, NOD1-deficient mice and PBMCs demonstrated enhanced joint inflammation and proinflammatory cytokines, respectively, upon infection [80].

#### 3.2.2. NLRP Inflammasome Proteins

NLRs like NLRP1 and NLRP3 constitute large multiprotein complexes called inflammasomes that contribute to cytosolic PGN sensing in response to pathogenic PAMPs (Figure 4). The inflammasome formation is initiated by several cytosolic PRRs, including NLRs (NLRP1 and NLRP3), AIM2-like receptors (ALRs) that detect foreign double-stranded DNA in the cytosol, the TRIM family protein pyrin, which acts as a sensor for toxin-induced cytoskeletal modifications, and RIG-I-like receptors (RLRs) that majorly sense viral RNA particles [81,82]. NLRP1 and NLRP3 also recognize slightly different ligands; NLRP1 binds MDP and primarily senses bacterial toxins, most notably the anthrax lethal factor, viral enzymes such as the 3C protease of enteroviruses, and ribotoxins [83,84], whereas NLRP3 is a more broad-spectrum sensor, capable of responding to ionic shifts in intracellular calcium and potassium levels, and metabolic or environmental stress [85] (Figure 4). This heterogeneity in PGN sensing by extracellular and intracellular PRRs shapes diverse immune outcomes during infection.

Upon assembly, NLRP1 and NLRP3 activate caspase-1, which converts pro-IL-1β and IL-18 into their mature and active forms for release and downstream signaling [86] (Figure 4). IL-1β induction has been shown to be protective against various bacterial infections by driving immune cell recruitment to damaged and infected tissues [87,88], but it can also act deleteriously when produced in excessive amounts, acting as a double-edged sword [89]. Importantly, IL-1β production by dendritic cells has been linked to T-cell differentiation and activation, highlighting its role in inducing critical adaptive immune responses. IL-18 induces IFN- γ expression and acts as a co-stimulatory molecule driving adaptive immunity [90]. As illustrated in Figure 4, active Caspase-1 also drives a lytic, proinflammatory form of cell death known as pyroptosis by cleaving Gasdermin-D (GSDMD), which then forms pores in the plasma membrane. This allows intracellular pathogens to be expelled from their replicative niches and generates additional DAMPs that further amplify immune detection and signaling [91].

Previous research has shown that upon degradation of PGN within host phagosomes, N-acetylglucosamine (NAG) can be sensed non-canonically in the cytosol by mitochondria-associated hexokinase, a glycolytic enzyme. NAG binding to hexokinase promotes its dissociation from the outer mitochondrial membrane, initiating NLRP3 inflammasome assembly and subsequently activating caspase-1 to promote IL-1β production. This work highlighted that NAG is the minimal NLRP3 activating PGN motif required for its direct activation in the cytosol [92] (Figure 4).

#### 3.2.3. Muropeptide Transport to the Cytosol

For muropeptides to gain access to intracellular PRRs, they must first enter the host cytosol. One, well-characterized route involves transmembrane peptide transporters of the SLC15A family, most notably PepT1 (SLC15A1), which are commonly expressed on the plasma membrane and on endosomal compartments [93]. These transporters enable the entry of small PGN fragments, including MDP and other PGN-derived ligands, from the extracellular space into the cytoplasm. SLC46A2, another cell-membrane transporter and solute carrier, is known for its role in shuttling muropeptides, specifically DAP type monomeric PGNs, including anhydromuropeptides, across the cell membrane [94] (Figure 4). Bacterial PGN is also taken up by host cells during cell wall remodeling through endocytosis and micropinocytosis. This process allows the cell to sample extracellular contents and concentrate PGN fragments within endosomal compartments so that they can subsequently be released into the cytosol.

Vesicle-based delivery systems like outer-membrane vesicles (OMVs) are also commonly used for constitutive shedding of PGN in gram-negative bacteria (Figure 4). These lipid bilayer-enclosed vesicles can fuse with host cells to deliver cargo into the cytosolic compartment, bypassing the need for specialized transport machinery [95]. More targeted delivery of PGN can sometimes occur through specialized secretion systems. The type IV secretion system in *H. pylori* acts as a molecular syringe that can directly deliver peptidoglycan fragments into the host cell cytosol, bypassing the need for phagocytosis or receptor-mediated endocytosis. This PGN is then sensed by NOD1, inducing downstream signaling pathways. Pathogens capable of intracellular invasion, like *L. monocytogenes*, directly breach the vacuolar membrane releasing PGN fragments into the cytosol and initiating NOD1/NOD2-mediated immune responses [96].

## 4. Peptidoglycan as a Toxin

### 4.1. Generation of Toxic PGNs

Despite its role as an inflammatory molecule, capable of stimulating host PRRs, PGN’s role as a potential toxin is less evident in the literature. Emerging research suggests that PGN is also capable of acting as a potent inducer of severe and toxic inflammatory responses, making it more than a by-product of cell wall remodeling. The toxic properties of gram-negative bacterial cell walls are mostly attributed to their outer membrane lipopolysaccharide LPS, an endotoxin that is released during growth and division, via OMVs and upon bacterial death [97,98]. In gram-positives, lipoteichoic acid (LTA), an immunostimulatory endotoxin-like cell wall polymer, takes this place [99]. However, very few studies have characterized PGN and its varied structural moieties as toxins in their own rights, independent of the contributions of LTA and LPS. Herein we review the limited literature exploring PGN as a toxin and synthesize how these diverse PGN structures shape immune detection or avoidance to promote pathogenesis.

Defective PGN recycling is a common yet immunologically critical phenomenon in bacteria that can drive the production of toxic, 1,6-anhydro-MurNAc-containing PGN fragments, arising from either antibiotic inhibition, genetic mutations, or regulatory failures [39]. A mechanistically distinct cause of PGN recycling failure is the impairment of inner membrane permeases responsible for reimporting anhydromuropeptides from the periplasm into the cytoplasm [100] (Figure 3). Excessive or unregulated LT activity can also overload the downstream recycling machinery thereby flooding the periplasmic space with anhydromuropeptides. This is usually accompanied by periplasmic overflow of LT products that exceed AmpG’s transport capacity, leading to extracellular fragment release [101] (Figure 3).

### 4.2. Toxic PGNs in Infection and Disease

#### 4.2.1. 1,6 Anhydromuropeptides

Muropeptides function as potent virulence factors in the pathogenesis of different human pathogens such as *Streptococcus pneumoniae*, *Bordetella pertussis*, *Neisseria gonorrhoeae*, *Listeria monocytogenes*, and *Helicobacter pylori* [102]. Many PGN monomers, including the potent toxin, tracheal cytotoxin (TCT), released by *B. pertussis* and *N. gonorrhoeae* possess a range of biological effects including cytotoxicity. Mutations in the AmpG promoter sequence of *B. pertussis*, as described in Section 2.3 of this review, result in over 95% TCT release into the extracellular environment [103,104] (Figure 3). TCT is a 921 Da GlcNAc-1,6-anhydro-MurNAc monomeric fragment consisting of a meso-DAP containing tetrapeptide chain, which has been shown to be critical for recognition by host NOD1 [105]. Structure activity data from the same study also revealed that the disaccharide moiety is dispensable for toxicity, whereas the free amino and carboxyl groups of the DAP- side chain retain biological and cytotoxic properties [105]. The 1,6-anhydro-N-acetylmuramic acid (anhMurNAc) residue is the defining structural motif that marks TCT and related cytotoxic PGN fragments as products of LT activity, distinguishing them from accidental peptidoglycan shedding. Following cleavage of the MurNAc-GlcNAc glycosidic bond, LTs catalyze the intramolecular cyclization between C1 and C6 of MurNAc, producing a non-reducing anhydro ring that is resistant to further degradation by host lysozyme [106,107] (Figure 1).

#### 4.2.2. TCT Release by *B. pertussis* and *N. gonorrhoeae*

Upon release by *B. pertussis* and *N. gonorrhoeae*, TCT enters the cytosol and binds NOD1, which triggers downstream NF-κB-mediated transcription of pro-inflammatory cytokines like IL-1α and IL-1β [108]. TCT has also been reported to induce the production of inducible nitric-oxide synthase (iNOS) in hamster tracheal epithelial cells and drive nitric oxide induction in response to pertussis infection leading to the characteristic destruction of ciliated epithelial cells in the trachea [109]. The loss of ciliated epithelium results in excess mucus and cellular debris that accumulate in the airways. In 3D human airway models, TCT alone was shown to drive blebbing and necroptosis of ciliated epithelial cells [110]. TCT also shares structural identity with Factor S, a neurologically active slow-wave sleep promoter and pyrogen isolated from human cerebrospinal fluid and urine [103]. As in *B. pertussis*, the immune outcomes of TCT release in *N. gonorrhoeae* are substantial. Gonococcal anhydro muropeptides induce the production of IL-1, IL-6, and IL-8 from human monocytes and epithelial cells via NOD1 activation, and this has been shown to drive the clinical symptoms of urethritis, cervicitis, and pelvic inflammatory disease [111].

#### 4.2.3. Orn and G-G-anhM-Containing Muropeptides in Lyme Disease

*Borrelia burgdorferi*, the spirochete that causes Lyme disease, produces an unusual peptidoglycan layer that sets it apart from typical gram-negative bacteria and contributes directly to its pathogenic persistence. *B. burgdorferi* PGN carries two striking structural anomalies (Figure 3). The first is a GlcNAc-GlcNAc-anhMurNAc (G-G-anhM) trisaccharide at the reducing terminus of its glycan strands, a deviation from the universal GlcNAc-MurNAc disaccharide repeat [112]. These soluble anhydromuropeptides are produced by the action of membrane-bound lytic transglycosylases (MltS) [113] and confer greater conformational flexibility to the peptidoglycan sacculus, making it significantly more resistant to host defenses [112]. The second is the substitution of L-ornithine for mDAP at position 3 of the stem peptide, which makes *B. burgdorferi* PGN a less potent NOD1 agonist. Clinically this PGN persists in the synovial fluid of patients with antibiotic-refractory Lyme arthritis long after antibiotic clearance of the spirochetes, sustaining inflammatory pathology and highlighting the role of anhM motifs in driving bacterial pathogenesis and persistence. Notably, this persistence of the PGN correlated with local antibody responses in the synovial fluid and Lyme arthritis symptoms, highlighting its immunogenicity and potential contribution to disease [48].

### 4.3. PGN-Mediated Immune Evasion

#### 4.3.1. PGN Enzymatic Modifications Driving Immune Evasion

**Figure 5:**
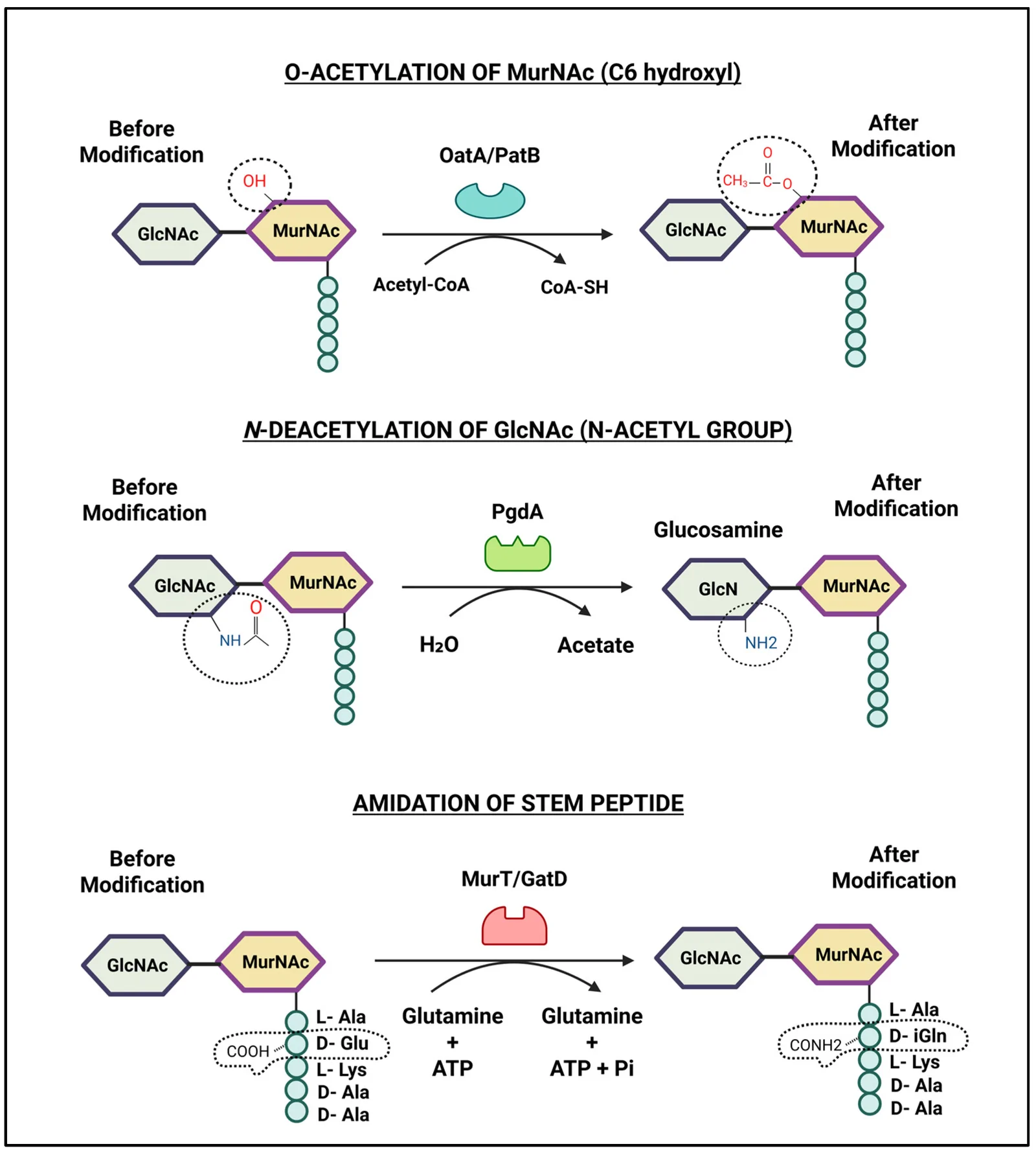
Enzymatic modifications of bacterial PGN. O-Acetylation of the C6 MurNAc hydroxyl group by enzymes OatA/PatB, which transfers an acetyl to MurNAc, releasing CoA-SH (**top**). N-Deacetylation of the GlcNAc N-acetyl group at position 3 by the deacetylase PgdA releasing acetate and resulting in glucosamine (GlcN) (**middle**). Amidation of the stem peptide by MurT/GatD, converting the carboxyl group of D-glutamate (D-Glu) to an amide (D-isoglutamine, D-iGln), using glutamine and ATP hydrolysis (**bottom**). [Created in BioRender. Cardozo, S. (2026) https://BioRender.com/e5ej5sr].

PGN undergoes various chemical modifications that enhance its ability to evade critical immune responses. One common example of PGN backbone modification includes *O*-acetylation of MurNAc at the C-6 hydroxyl group, which is carried out by the integral membrane acetyltransferase OatA in gram-positives or the two-component OatB/PatB system in gram-negatives [114] (Figure 5). This modification, which involves transferring an acetyl group from acetyl CoA onto the saccharide backbone, sterically impairs recognition by host lysozyme, a key host defense enzyme, thereby conferring resistance to enzymatic degradation and allowing pathogens like *S. aureus* and *N. gonorrhoeae* to persist in host environments [115,116].

Another, chemically distinct sugar backbone modification includes N-deacetylation, which is conducted by enzymes such as PgdA, a metal-dependent esterase in *S. pneumoniae* and *L. monocytogenes*, and IcaB, a de-N-acetylase enzyme in *S. epidermidis* and *H. pylori* remove the N-acetyl group from GlcNAc residues in ꞵ-1,6-linked N-acetylglucosamine polymers (PNAG) respectively [117] (Figure 5). Removal of the N-acetyl group reduces susceptibility to host lysozymes and pattern recognition receptors like NOD1 and NOD2, allowing for bacterial immune evasion [118,119]. Deletion of PgdA and IcaB have also been shown to impair bacterial virulence and biofilm formation [117,120].

The stem peptide also undergoes chemical modifications that enhance bacterial pathogenesis in the host. As outlined in Figure 5, amidation of the α-carboxyl group of D-glutamate (at position two of the stem peptide) to D-isoglutamine in most gram-positive bacteria, or amidation of the meso-diaminopimelic group (mDAP) carboxylic group in gram-negatives is catalyzed by enzymes such as MurT and GatD that make up the amidotransferase complex. GatD hydrolyzes glutamine to generate ammonia, which is then channeled to the active site of MurT to be used for amination of the PGN stem [121,122]. This reduces the overall negative charge of the bacterial cell wall, driving resistance to host lysozymes [123].

#### 4.3.2. Non-Canonical PGN Structures That Promote Immune Evasion

Aside from post-synthetic PGN modifications, non-canonical bacterial muropeptides generated during PGN synthesis, as described in Section 2.3 and Section 4.2, can also contribute to immune evasion. The energetically expensive shedding of bacterial anhMurNAc-bearing PGN fragments suggests that the advantages of generating these immunomodulatory muropeptides might outweigh the metabolic investment.

We have recently shown that TCT release from *B. pertussis* skews the immune response toward a less inflammatory phenotype by promoting NOD1 and suppressing NOD2 activation. This is potentially due to recognition by NOD2 requiring access to the reducing-end anomeric configuration of MurNAc, which is hindered by the presence of 1,6 anhydro moieties [124]. We also show that NOD2-driven IL-1β production in myeloid cells activates airway fibroblasts to secrete CXCL13 and CCL19 that could potentially recruit B cells and CD4+ T cells for adaptive clearance. TCT-mediated suppression of this axis allows the bacteria to persist [125]. Additionally, TCT dampens TREM-1 activation, preventing early inflammatory signaling responses to escape immune detection and compromising critical long-term adaptive immunity [58]. *N. gonorrhoeae* achieves a similar outcome through its two dedicated lytic transglycosylases, LtgA and LtgD, which over-produce the 1,6-anhydro monomers that were observed to be poor NOD2 agonists. When these enzymes are deleted, NOD2 activation increases substantially, highlighting that LtgA/LtgD activity itself could be an immune evasion strategy that limits NOD2 engagement [126] (Figure 3).

The probiotic *Bifidobacterium* species displays an extraordinarily high abundance of 1,6-anhydro-MurNAc (anNAM)—containing PGNs—up to 40% of total PGNs in *B. adolescentis*, which is unusual for Gram-positive bacteria [124]. Crucially, these *Bifidobacterium* anhydro-PGNs are structurally incapable of activating either NOD1 (due to the L-Lys or L-Orn in the stem peptide instead of mDAP) or NOD2, because the 1,6-anhydro bond blocks recognition. A similar dual evasion strategy is seen in the pathogen *Borrelia burgdorferi*, which is able to leverage its DAP-to-L-ornithine modification to dampen NOD1 activation [63], while the 1,6-anhydro-MurNAc fragments generated by its LT (MltS) are poor NOD2 agonists [113]. Another comparable stem-peptide modification occurs in *M. leprae* and *C. trachomatis*, where glycine replaces L-alanine at the first stem-peptide position through the activity of MurC ligases [127,128]. Unlike the evasion strategies described above, this altered PGN peptide stem, still activated NOD2 comparable to canonical MDP [129]; however, its potential impact on NOD1 recognition and other PGN-sensing PRRs remain untested.

## 5. Conclusions

PGN has long been viewed as a structural scaffold for bacterial cell wall strength and durability. Here we discuss the literature that identifies PGN as a PAMP whose structural features are actively interpreted as diverse cues by host PRRs to drive critical immunity [130,131]. The selectivity of PRRs for specific PGN motifs has emerged as an area of growing interest [132]. Receptors such as NOD1 and NOD2 sense PGN fragments based on terminal amino acids and disaccharide structures, while peptidoglycan recognition proteins such as PGLYRP1 engage specific glycopeptides to activate downstream signaling pathways [66,133]. This highlights differences in the immune recognition potential of these extracellular and cytosolic PRRs that allow us to uncover diverse PGN motifs and their preferred PRR responses.

We provide a novel overview of how PGN fragments can become toxic molecular stimuli for the host. Strategic modifications to PGN, either post synthesis or during remodeling reshape the pool of fragments that the immune system encounters, shifting some PGN species from inert recycling by-products into toxic danger signals. Of all the structural modifications that shape PGN fragment fate, the 1,6-anhydro bond at the reducing end of muramic acid, as discussed in this review, has emerged as a significant determinant of immune recognition and bacterial immune evasion. From the bacterial perspective, it is unclear why bacteria have evolved to shed this non-canonical structure, risking both fitness costs and immune detection in the host. The most plausible reason for this from the host viewpoint is that NOD2 activation requires prior phosphorylation of the MurNAc C6 hydroxyl group by the mammalian kinase NagK [134]. However, this 1,6-anhydro modification converts C5 and C1 into a bicyclic ring that lacks the free C6 hydroxyl group, impairing its availability as a NAGK substrate and thereby rendering it entirely invisible to NOD2 [113].

Recent studies elucidate a striking divergence in NOD1 and NOD2 signaling pathways in response to infection, with NOD1 emerging as a less potent driver of inflammation compared to NOD2. This partially explains why the release of these non-canonical anhydromuropeptides might be strategic and intentional rather than random or erroneous, suggesting the failure to recycle these PGN fragments as seen in *B. pertussis*, *N. gonorrhoeae,* and *Borrelia burgdorferi* may be beneficial adaptations. These examples of structure-based immune avoidance reflect how bacteria have evolved to use metabolic failures and non-canonical PGN release in their favor.

Ultimately the diversity of host bacterial PGN ligands and their respective PRR-mediated immune outcomes reflect their ability to preferentially drive immunoinflammatory or protective responses highlighting their role as immunomodulators. These defined muropeptides with known receptor preferences are promising candidates for adjuvants in next-generation designer vaccines that drive effective immune responses. Studies that shed light on how these structurally defined muropeptides emerge and why they preferentially engage certain host receptors, will provide more mechanistic data on their immunomodulatory roles. This review details how peptidoglycan functions as a structurally diverse pool of ligands dictating host-pathogen interaction. Specifically, our review highlights the role of PGN as a critical cue for immune detection and activation, with potential consequences for vaccine and therapeutic development.

## Data Availability

No new data were created or analyzed in this study. Data sharing is not applicable to this article.

## Abbreviations

The following abbreviations are used in this manuscript:

PGN	peptidoglycan
PRR	pattern recognition receptor
NLR	NOD-like receptor
ASC	apoptosis-associated speck-like protein containing a CARD
ATP	adenosine triphosphate
dsDNA	double-stranded DNA
ERK	extracellular signal-regulated kinase
GlcN	glucosamine
GlcNAc	N-acetylglucosamine
GSDMD	gasdermin D
IκB	inhibitor of kappa B
IL	interleukin
MDP	muramyl dipeptide
mDAP	meso-diaminopimelic acid
MurNAc	N-acetylmuramic acid
NF-κB	nuclear factor kappa-light-chain-enhancer of activated B cells
OMV	outer membrane vesicle
ORN	ornithine
TCT	tracheal cytotoxin
Th17	T helper 17 cell
TNF-α	tumor necrosis factor alpha
Treg	regulatory T cell
UDP	uridine diphosphate
